# Machine learning prediction of depression in culturally diverse families: Findings from the Korea Community Health Survey

**DOI:** 10.3389/fpubh.2025.1666084

**Published:** 2025-09-18

**Authors:** Geun Myun Kim, Sunkyung Cha, Miran Jung, SeongKwang Kim

**Affiliations:** ^1^Department of Nursing, Gangneung-Wonju National University, Wonju, Gangwon-do, Republic of Korea; ^2^Department of Nursing Science, Sun Moon University, Asan, Chungcheongnam-do, Republic of Korea; ^3^Department of Nursing, Baekseok University, Cheonan, Chungcheongnam-do, Republic of Korea

**Keywords:** depression, machine learning, XGBoost, cultural diversity, Republic of Korea

## Abstract

**Background:**

Although South Korea's overall population is declining, the number of culturally diverse families is increasing. Depression in these families is a significant factor contributing to rising social costs and hindering social integration.

**Methods:**

To predict depression in culturally diverse families in South Korea, we analyzed 131 independent variables from 2,568 culturally diverse families who participated in the 2023 Korea Community Health Survey.

**Results:**

We identified 15 key predictive variables and evaluated their effects using the XGBoost model, which outperformed 5 other machine learning models. Stress recognition, experience of extreme sadness or despair, subjective health status, age, and frequency of contact with neighbors emerged as significant predictive factors.

**Conclusions:**

By conducting a comprehensive analysis of multidimensional indices, this study offers a multifaceted perspective on depression in culturally diverse families.

## 1 Introduction

With the accelerating pace of globalization, the number of culturally diverse families is growing worldwide. This trend is not limited to traditional immigrant-receiving countries such as the United States, Canada, and Australia but has also been reported in Europe and Asia (1–3). This trend is also evident in South Korea. In 2023, there were 415,584 culturally diverse households in South Korea, representing an approximate 4% increase from the previous year. Since 2020, the number of culturally diverse families has grown annually by 3.7–4.7%, and their share of the total population has risen from 1.7% in 2015 to 2.3% in 2023 (4).

Culturally diverse families are families characterized by differences in beliefs, practices, languages, and values due to cultural differences either among family members or between the family and the surrounding society (5). This concept includes families formed through international marriage and immigrant families. The rise in culturally diverse families has engendered various challenges related to social integration and adaptation (6). Members of these families often experience high rates of depression due to language barriers, economic difficulties, social prejudice and discrimination, and cultural differences (6–10). Culturally diverse families that include racial minorities are at a higher risk of depression than culturally homogeneous families (7–9). Additionally, immigrants, including refugees, have been reported to show a high incidence of depression (9), and studies in South Korea have found that depression scores are higher among culturally diverse family members than among those in culturally homogeneous families (11). Beyond individual distress, depression in culturally diverse families contributes to increased social costs and hinders social integration (6–10).

Previous studies have identified various personal, familial, and social factors that influence depression in culturally diverse families. Personal factors include age, gender, education, language proficiency, self-esteem, social withdrawal, physical activity, and health behaviors (8, 10–13). Family-related factors such as parental age, economic status, parenting attitudes, family communication method, spousal relationships, and overall family dynamics have also been linked to depression risk (7, 8, 10, 12–15). Additionally, social and environmental factors—including area of residence, participation in economic activities, local social networks, and experiences of discrimination—have been shown to impact depression levels in culturally diverse families (7, 8, 10, 13–15).

However, the impact of these factors varies across countries depending on their sociocultural context. Understanding depression in culturally diverse families requires a country-specific approach that accounts for these sociocultural influences (16). South Korea presents a unique case in this regard. Its social characteristics and the composition of culturally diverse families differ from those in other immigrant-receiving countries. In countries such as the United States and Canada, immigrants come from a wide range of backgrounds, and because diverse ethnicities and races coexist within these societies, culturally diverse families are perceived as a common and natural part of the social fabric (17). Contrastingly while some culturally diverse families in South Korea are formed through naturalization, more than 80% result from marriages between a Korean national and a foreign spouse (12). Furthermore, South Korea's strong emphasis on bloodlines and ethnic homogeneity leads to a relatively low level of acceptance of culturally diverse families (10). This social environment fosters greater prejudice and discrimination against members of these families (6), poses significant challenges to community integration and mental health, and serves as a major contributing factor to depression within these families (18). Given that perceptions of culturally diverse families in South Korea differ from those in other countries, it is essential to analyze depression within this specific context and identify its key contributing factors. This task is particularly urgent given the increasing presence of culturally diverse families in South Korean society.

Much of the recent research on culturally diverse families in South Korea has focused on children and adolescents (10, 11, 14–16). However, when identifying predictive factors for depression, it is also crucial to consider the role and psychological influence of adults in the family. Research on adults remains relatively scarce, despite its importance. Furthermore, to accurately assess the factors influencing depression in culturally diverse families, comparisons with culturally homogeneous families are necessary. As depression in culturally diverse families is influenced by a wide range of factors, it is also important to examine variables that have not been previously identified.

One valuable source of data for exploring depression and related variables in culturally diverse families in South Korea is the 2023 Korea Community Health Survey (KCHS). The KCHS dataset consists of recent data collected from approximately 230,000 adults aged 19 years or older in South Korea. The survey includes over 140 items covering health behaviors such as physical activity and diet, and the management of chronic diseases, including mental health disorders and hypertension. Data collection was conducted at 258 survey centers nationwide.

However, the broad scope of the survey imposes clear limitations on analysis using conventional methods such as regression. These limitations include difficulty in fully capturing complex relationships between variables and the potential for missing important predictors. Recent studies have highlighted these limitations and reported that machine learning (ML) models demonstrate significantly superior predictive performance compared to conventional approaches such as logistic regression (LR) (19–22). In particular, research comparing ML and LR on datasets containing complex and diverse variables—such as EMR-based predictive models for 30-day readmission in heart failure patients (19), predictive models for myocardial illness (20), and models predicting severe COVID-19 in hospitalized children infected with the Omicron variant (21)—has shown that ML consistently outperforms LR. Similarly, a study on predicting depression in adults aged 18 years and older has reported that ML, particularly XGBoost, achieved higher accuracy, sensitivity, specificity, precision, AUC, and F1 scores compared to LR (22).

Thus, Machine learning (ML) ensemble models not only offer a way to address these challenges but also improve prediction performance and stability by combining multiple single-tree models, reducing overfitting, and enhancing generalization ability. Additionally, these models account for non-linear relationships and interactions between multiple variables (23). Moreover, SHapley Additive exPlanations (SHAP) can be used to analyze the importance of each variable (24), making these models more applicable to policymaking for mental health support in culturally diverse families.

In this study, we implemented an ML ensemble model using data from the 2023 KCHS to predict depression in culturally diverse families, identify key contributing variables, and provide evidence to support more effective mental health policies.

## 2 Methods

### 2.1 Study design

This study is an ML-based secondary data analysis aimed at predicting depression in culturally diverse families using data from the 2023 KCHS (https://chs.kdca.go.kr/chs/index.do).

### 2.2 Data collection

We utilized data from the 2023 KCHS, a nationwide survey conducted by the Korea Disease Control and Prevention Agency in 255 cities, counties, and districts. The KCHS is a cross-sectional survey that employs stratified cluster sampling and collects data on health status, behaviors, and healthcare utilization.

The sample was selected using probability proportional to size sampling based on resident registration and housing data. Trained surveyors conducted face-to-face household interviews using the Computer-Assisted Personal Interviewing method to ensure data completeness without missing responses.

The KCHS survey on culturally diverse families has been conducted every 3 years since 2019, most recently in 2023. The KCHS defines culturally diverse families as those composed of individuals from diverse national, ethnic, and cultural backgrounds. This includes families with at least one member who has immigrated to South Korea, even if they have acquired Korean nationality; families in which the respondent was born in South Korea but has at least one parent born overseas; and individuals who self-identify as belonging to a culturally diverse family. The KCHS provides a representative and reliable national-scale dataset. The dataset used in this study was publicly available and anonymized. This study involved a secondary analysis of that dataset. Prior to data collection and analysis, the study was granted an exemption from ethical review by the institutional review board of the university (GWNUIRB-R2025-10), because it utilizes publicly available anonymized data.

### 2.3 Methods of data analysis

**Figure 1:**
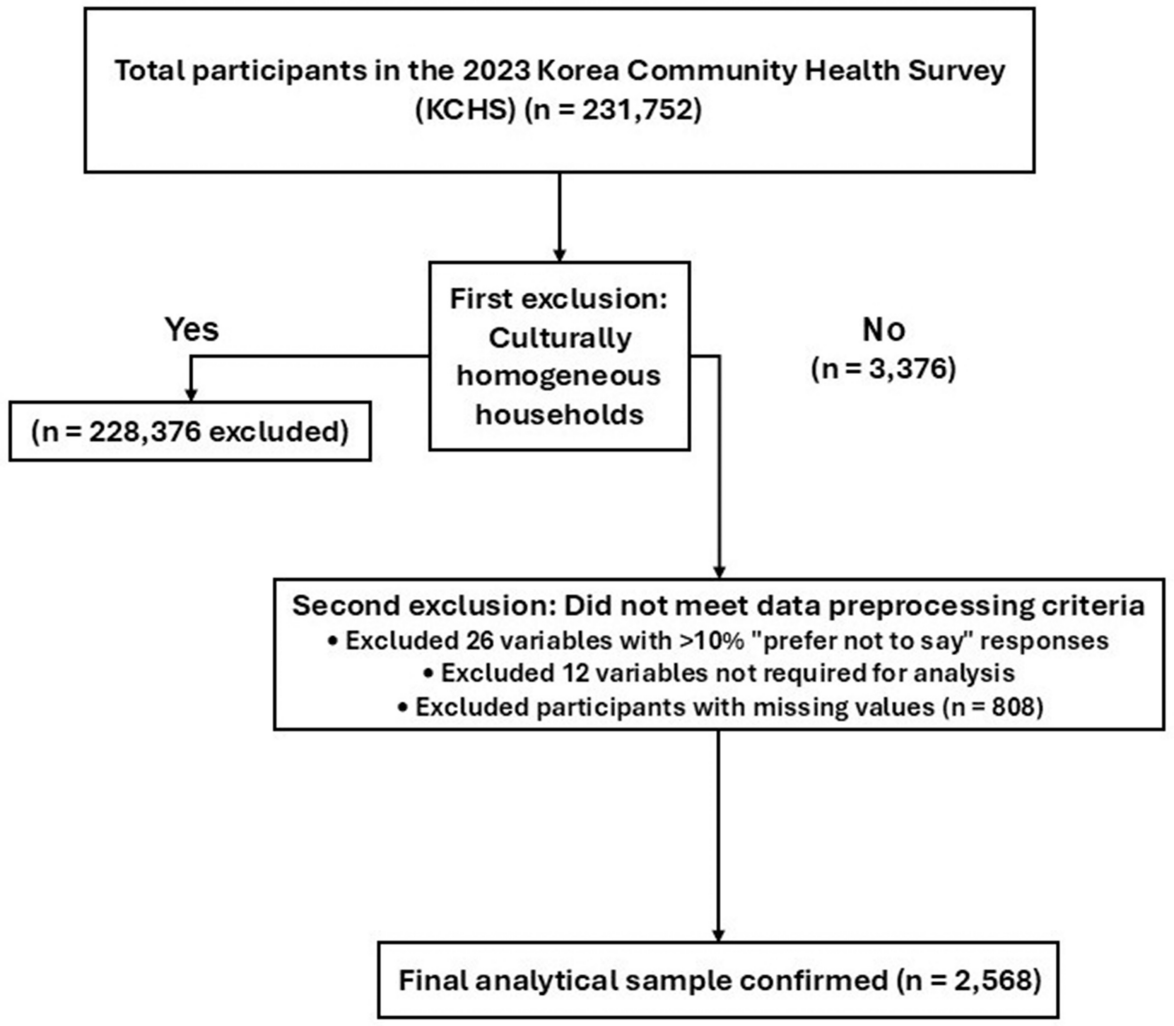
Research process for predicting outcomes using ML models.

We used Python (version 3.9, Python Software Foundation, USA) to preprocess the data and construct the ML models in this study. The data analysis pipeline included data collection, data preprocessing, exploratory data analysis, model construction and training, performance evaluation, optimal model selection, and results interpretation (Figure 1).

#### 2.3.1 Data processing

The data analyzed in this study consisted of 17 total categories, 178 items, and 231,752 participants (culturally diverse group: 3,376 individuals; culturally homogeneous group: 228,376 individuals). For this study, only the data from 3,376 individuals belonging to culturally diverse families were included in the analysis. During data preprocessing, we established criteria for processing specific responses to ensure consistency and reliability. First, we excluded 26 variables where the proportion of “prefer not to say” responses exceeded 10% across the entire sample. For variables with a “prefer not to say” response ratio below 10%, such responses were treated as anomalies and removed, and the remaining data were included in the analysis (25). However, responses of “not sure” were considered meaningful, as they could reflect the sociocultural context of culturally diverse families, and they were therefore retained in the analysis.

Additionally, we excluded 12 variables that were not required as independent variables, such as the survey year, sample index, public health center, stratification and cluster weight variables, and the variable indicating culturally diverse family status (this variable was removed after retaining only individuals from culturally diverse families). We derived the Patient Health Questionnaire-9 (PHQ-9; mtb_07a1 to mtb_07i1) as the dependent variable and excluded the original mtb_07al to mtb_07il from the independent variables.

**Figure 2:**
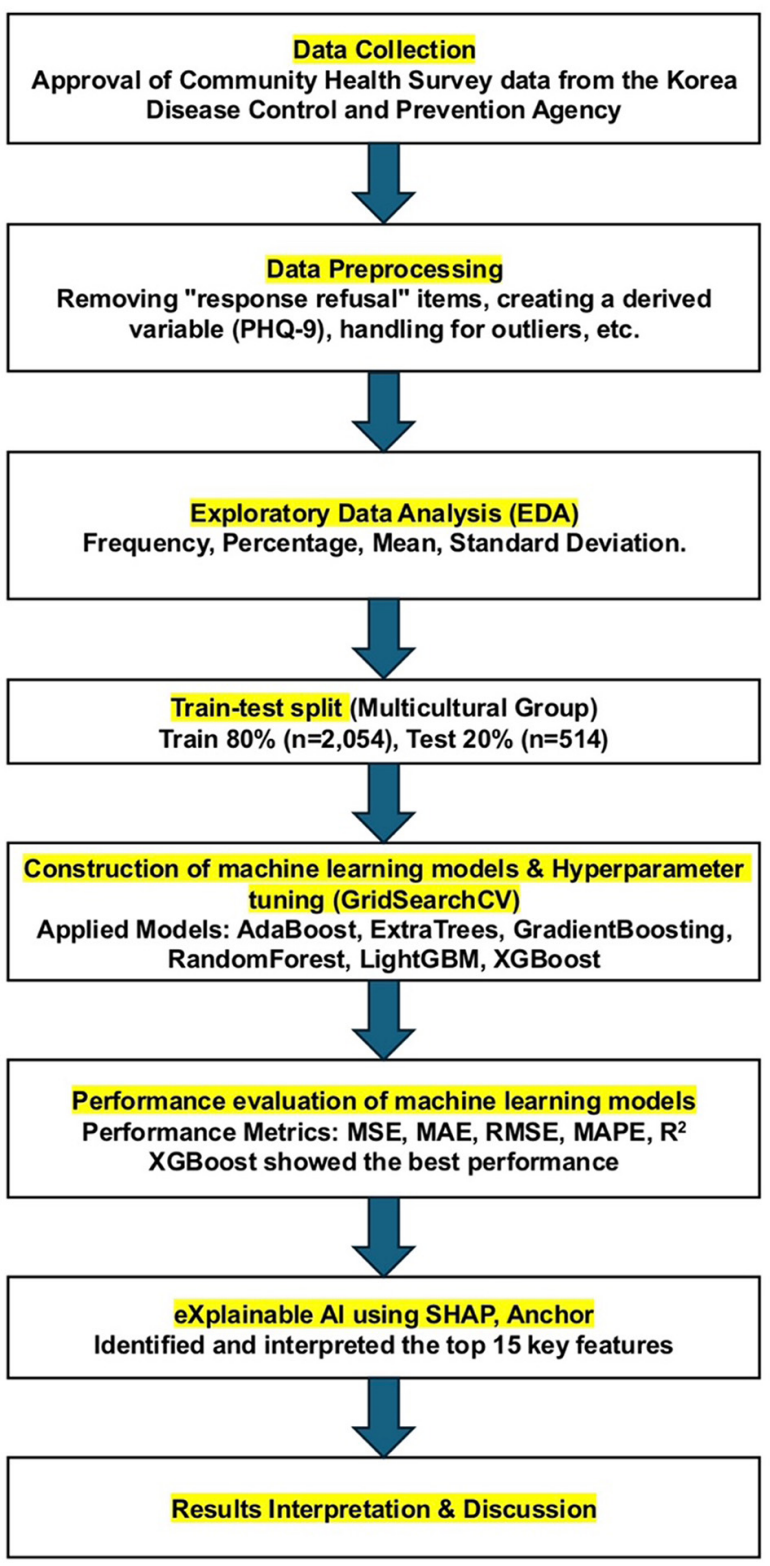
Flowchart of the participant selection process.

The final dataset included 2,568 participants from culturally diverse families. All 131 available independent variables were included without selection to ensure model comprehensiveness, along with one dependent variable. A flowchart of the participant selection process is presented in Figure 2.

We deliberately included all 131 variables without a preliminary feature selection or reduction step for two primary reasons based on the strengths of our chosen modeling approach.

First, the tree-based ensemble models used in this study, such as RandomForest (26) and XGBoost (27), are inherently robust to multicollinearity. Unlike regression models where correlated predictors can lead to unstable coefficient estimates, tree-based models mitigate this issue during the splitting process (26–28). If two variables contain similar information, the algorithm will typically select one for a split, effectively downweighting the redundant variable without negatively impacting the model's overall predictive performance. Therefore, a separate diagnostic procedure for multicollinearity (e.g., calculating Variance Inflation Factor) was not necessary (26–28).

Second, these models perform a form of intrinsic feature selection. Variables that are not informative are simply not chosen for splits during the tree-building process and consequently receive very low importance scores (26–31). By including all available variables, we allowed the model to explore the entire feature space for complex, non-linear relationships that a preliminary feature reduction step might have obscured. This approach aligns with our goal of conducting comprehensive, data-driven analysis to ensure model comprehensiveness.

#### 2.3.2 Exploratory data analysis of the main variables

Exploratory data analysis was performed to characterize the main variables. For categorical variables, frequencies and percentages were calculated to examine their distributions. For continuous variables, means and standard deviations were computed to assess central tendency and dispersion. Furthermore, skewness and kurtosis were evaluated to assess the normality and distributional properties of continuous variables.

#### 2.3.3 Train-test data split

To predict depression in culturally diverse families, we split the culturally diverse group dataset (*n* = 2,568) into 80% training data (*n* = 2,054) and 20% test data (*n* = 514). To ensure model stability and generalizability, we performed a five-fold cross-validation. This process involves dividing the training data into five subgroups and sequentially validating each one, thereby helping to prevent overfitting and improve model reliability.

#### 2.3.4 ML model construction and hyperparameter tuning

##### 2.3.4.1 ML model selection

For this study, we deliberately focused exclusively on tree-based ensemble algorithms. This focused approach allows for a more direct and methodologically consistent comparison of state-of-the-art algorithms. We applied six ensemble algorithms to predict depression in culturally diverse families: RandomForest, XGBoost, LightGBM, Gradient Boosting, AdaBoost, and ExtraTrees. Compared to single models, ensemble learning enhances predictive accuracy and generalizability by combining multiple weak learners (26–28). In particular, tree-based ensemble models offer advantages such as improved variable importance evaluation, overfitting prevention, and efficient handling of large datasets (29–31). Additionally, these models reduce prediction errors through loss function optimization and regularization while controlling model complexity to maintain stable performance across diverse environments.

In this study, we compared the six models based on their predictive performance and interpretability to select the most suitable model for predicting depression in culturally diverse families.

##### 2.3.4.2 Hyperparameter tuning for the predictive models

Hyperparameters are key parameters that control model learning and must be defined by the investigator before training, as they significantly influence model performance (32). To optimize each ML algorithm, we applied the GridSearchCV method, which systematically evaluates combinations of hyperparameters within predefined ranges to balance efficiency and performance.

In this study, key hyperparameters tuned included max_depth, learning_rate, min_samples_split, n_estimators, min_samples_leaf, max_features, subsample, colsample_bytree, num_leaves, min_data_in_leaf, and loss. Specifically, max_depth determines the maximum depth of decision trees; learning_rate regulates the contribution of each tree in boosting models; min_samples_split and min_samples_leaf control the minimum samples required to split a node and to form a leaf, respectively; n_estimators specifies the number of trees or boosting iterations; and max_features defines the proportion of features considered at each split. For boosting algorithms, subsample and colsample_bytree adjust the fractions of samples and features used to build each tree, respectively, thereby controlling overfitting. In LightGBM, num_leaves and min_data_in_leaf govern the complexity and regularization of the tree structure. Finally, loss defines the loss function to be optimized in AdaBoost (e.g., linear, square, or exponential).

#### 2.3.5 Predictive model evaluation

##### 2.3.5.1 Indices for performance comparison

We evaluated the performance of the regression models using mean squared error (MSE), mean absolute error (MAE), root mean squared error (RMSE), mean absolute percentage error (MAPE), and *R*^2^. The formulas used to calculate these metrics are as follows:

- Mean Absolute Error (MAE) : $\frac{1}{n}\sum_{i=1}^{n}\left|y_{i}-\hat{y_{i}}\;\right|$.
- Mean Squared Error (MSE): $\frac{1}{n}\sum_{i=1}^{n}{\left(Y_{i}-\hat{Y_{i}}\;\right)}^{2}$.
- Root Mean Squared Error (RMSE): $\sqrt{\frac{1}{n}\sum_{i=1}^{n}{\left(y_{i}-\hat{y_{i}}\;\right)}^{2}}$.
- Mean Absolute Percentage Error (MAPE): $\frac{100}{n}\sum_{i=1}^{n}\left|\frac{y_{i}-\hat{y_{i}}}{y_{i}}\;\right|$.
- *R*-squared (*R*^2^): $1-\frac{\sum_{i=1}^{n}{\left(y_{i}-\hat{y_{i}}\right)}^{2}}{\sum_{i=1}^{n}{\left(y_{i}-y\;\right)}^{2}}$.

Where “*n*” is the total number of samples, “Σ” represents the summation over all samples, “y_i” is the actual value, “y_i” (y-hat) is the predicted value, and “ȳ” (*y*-bar) is the mean of the actual values.

Notably, MSE measures prediction accuracy by assigning greater weights to larger errors; MAE, while dependent on data scale, is advantageous due to its intuitive interpretation; RMSE, the square root of MSE, is similar to MAE but more sensitive to large errors; and MAPE calculates the average prediction error as a percentage of actual values, making it useful for evaluating relative error. However, these indices can theoretically extend infinitely beyond zero. Therefore, we primarily interpreted the results based on *R*^2^, using the other indices as complementary measures.

##### 2.3.5.2 SHAP

To assess feature importance, we applied a multifactorial approach. First, we generated an elbow plot using SHAP values for XGBoost, the model with the highest predictive power, and analyzed changes in predictive strength. Second, to enhance model interpretability, we selected features with SHAP values ≥0.05 (33). Finally, we reviewed the clinical validity of the selected features using domain knowledge from mental health nursing, incorporating insights from previous studies and nursing theory. Based on these criteria, we identified the top 15 features contributing to PHQ-9 score prediction.

Moreover, SHAP is a method for interpreting ML model predictions based on Shapley values, a concept from game theory. It quantifies the impact of each feature on model predictions. The sign of the SHAP value indicates whether a feature increases (positive) or decreases (negative) the predicted value.

In this study, we evaluated model performance by introducing features stepwise (34) and found that adding more than 15 features did not significantly improve performance. Therefore, based on SHAP values, we finalized the selection of the top 15 features with the most significant influence on depression prediction in culturally diverse families.

#### 2.3.6 Anchor method

To enhance the interpretability of the predictive model, we additionally applied the anchor method (35), a high-precision, model-agnostic explanation technique. Anchors are defined as sets of sufficient conditions (i.e., if-then rules) that locally “anchor” a model's prediction with high precision. Thus, an anchor represents the minimal set of feature constraints necessary for the model to consistently produce the same prediction with a high probability.

The anchor method can be applied to datasets containing categorical, binary, or continuous variables. For categorical or binary variables, anchors are expressed as specific feature-value pairs (e.g., “gender = female”). For continuous variables, conditions are generated through thresholding (e.g., “age > 65”) to define interpretable decision boundaries. In this study, as the dataset primarily consisted of categorical variables, categorical variables were used without modification, while continuous variables were discretized into quartile-based bins to construct anchors.

During the anchor search process, the minimum precision thresholds were set at 90% and 95%, respectively, to compare the sets of sufficient conditions and evaluate their impact on the stability of model predictions and reliability of the explanations. Additionally, 1,000 perturbation samples were generated per instance to evaluate the robustness and local consistency of the anchor conditions.

## 3 Results

### 3.1 Exploratory data analysis of main variables

In total, 2,568 participants were analyzed; the descriptive statistics for all variables are presented in Supplementary Table 1. Mental-health-related factors showed that 22.5% of respondents reported high perceived stress and 9.2% had experienced severe sadness or despair in the previous year, while the impact of digital media overuse was reported at least weekly by 6.0% of participants. Physical-health-related factors revealed that chewing discomfort was present in 19.8% of the sample, 15.5% rated their overall health as poor, and 52.1% had not engaged in any flexibility exercise during the past week. For personal factors, 37.7% of respondents perceived themselves as slightly or very overweight, 6.7% reported unmet medical needs within the past year, and 2.3% showed low adherence to handwashing after outings. Regarding social environmental factors, 58.9% resided in rural areas, 10.8% had been exposed to second-hand smoke in indoor public places during the past year, 33.1% consumed breakfast 4 or fewer times per week, and social interaction was limited with contact with neighbors once a month or less reported by 38.3% and contact with friends at the same frequency by 23.3%. The mean age of the sample was 50.55 ± 15.72 years, and the mean PHQ-9 score was 11.10 ± 2.96, indicating multiple individual and environmental risk factors that may contribute to depressive symptomatology in this population.

### 3.2 Model hyperparameters and performance evaluation

We constructed six types of ML models and used the GridSearchCV method to identify the optimal hyperparameters. To ensure model consistency, we set the bootstrap argument to “true” and the “random_state” argument to 42. We then performed a five-fold cross-validation to evaluate the generalizability of the models. The optimal hyperparameters for each model are presented in Supplementary Table 2.

Among the six models, XGBoost demonstrated the best performance on the test dataset. For the training dataset, XGBoost achieved an MAE of 1.65 (95% CI: 1.59–1.72), RMSE of 2.38 (95% CI: 2.23–2.54), MSE of 5.68 (95% CI: 4.94–6.42), MAPE of 14.0% (95% CI: 13.7–14.3%), and R^2^ of 33.9 (95% CI: 25.9–42.0). Performance on the test dataset was similar, with an MAE of 1.67, RMSE of 2.50, MSE of 6.23, MAPE of 14.0%, and R^2^ of 33.1%. These results indicate that model performance remained consistent between the training and test datasets (Table 1).

**Table 1:** Model performance evaluation metrics.

**Models**	**Training dataset**					**Test dataset**				
	**MAE (95% CI)**	**RMSE (95% CI)**	**MSE (95% CI)**	**MAPE (%) (95% CI)**	***R***^2^ **(%) (95% CI)**	**MAE**	**RMSE**	**MSE**	**MAPE (%)**	***R***^2^ **(%)**
Random Forest	1.71 (1.62–1.80)	2.43 (2.23–2.63)	5.94 (4.97–6.91)	14.6 (14.2–14.9)	31.2 (24.7–37.6)	1.72	2.51	6.32	14.5	32.1
Gradient Boosting	1.65 (1.59–1.72)	2.38 (2.24–2.52)	5.68 (5.02–6.34)	14.0 (13.7–14.3)	33.8 (25.9–41.8)	1.66	2.50	6.25	13.8	32.9
Extra Trees	1.72 (1.64–1.79)	2.45 (2.25–2.64)	6.01 (5.06–6.95)	14.6 (14.3–14.9)	30.4 (23.9–36.8)	1.71	2.50	6.25	13.8	31.5
AdaBoost	1.78 (1.69–1.86)	2.53 (2.31–2.75)	6.42 (5.32–7.53)	15.0 (14.5–15.5)	25.5 (16.3–34.7)	1.76	2.58	6.67	14.8	28.4
XGBoost (Best model)	1.65 (1.59–1.72)	2.38 (2.23–2.54)	5.68 (4.94–6.42)	14.0 (13.7–14.3)	33.9 (25.9–42.0)	1.67	2.50	6.23	14.0	33.1
LightGBM	1.65 (1.58–1.72)	2.38 (2.23–2.53)	5.67 (4.95–6.39)	13.9 (13.6–14.3)	34.1 (27.3–40.9)	1.68	2.51	6.31	14.1	32.3

### 3.3 XGBoost-based SHAP bar plot

**Figure 3:**
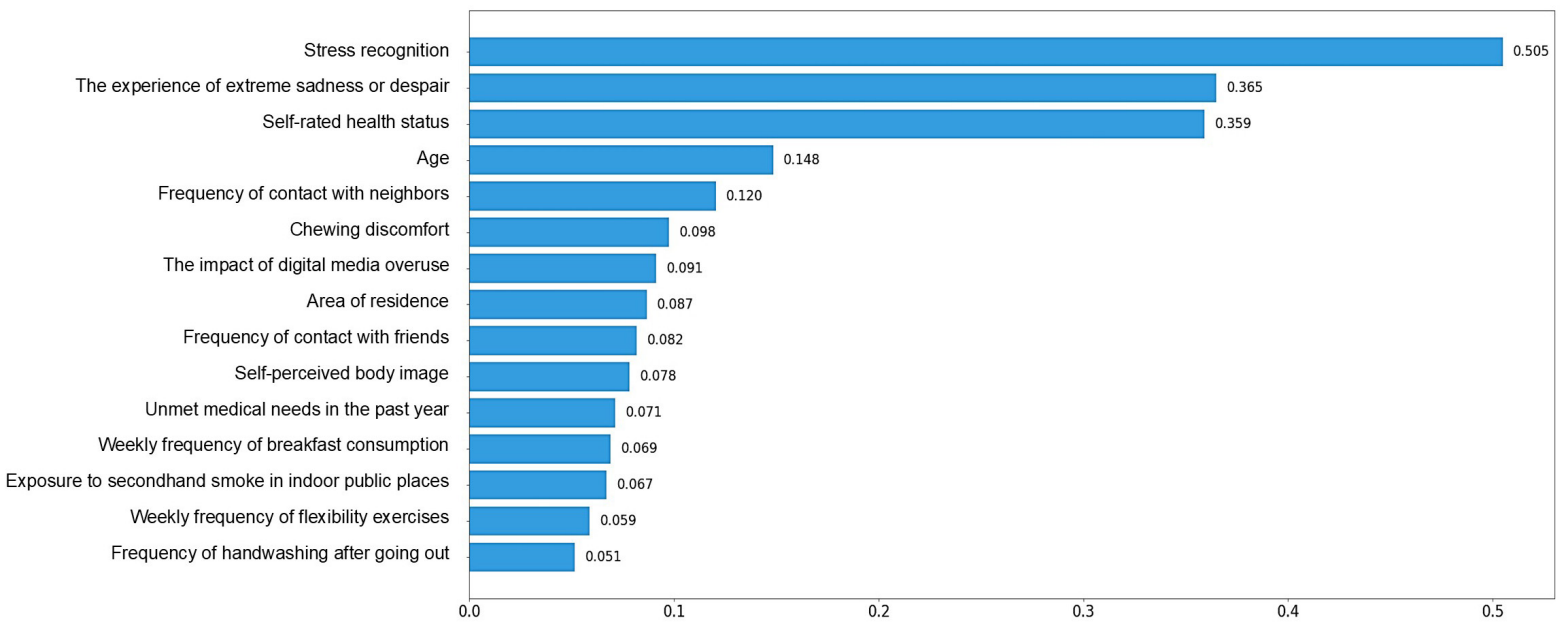
XGBoost-based SHAP bar plot.

When analyzing feature importance based on SHAP values for the top 15 features, stress recognition emerged as the most significant predictor, with a mean SHAP value of 0.51, indicating its significant impact on the model's predicted values. Other key features included the experience of extreme sadness or despair (mean SHAP = 0.37), subjective health status (mean SHAP = 0.36), and age (mean SHAP = 0.15). These features played crucial roles in shaping the model's predictions. Figure 3 illustrates the importance of the SHAP feature for each variable.

### 3.4 XGBoost -based SHAP summary plot

**Figure 4:**
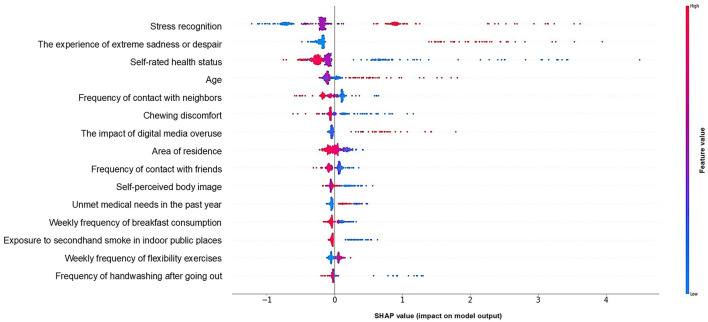
XGBoost-based SHAP summary plot.

We used a SHAP summary plot to visualize how features influenced model predictions. Higher values (red) for stress recognition and experience of depressive symptoms increased the model's output, indicating a higher likelihood of elevated PHQ-9 scores. Conversely, lower values (blue) reduced the predicted scores. For subjective health status and chewing discomfort, the trend was reversed: lower values (blue) increased the model output, while higher values (red) decreased it. This suggests that poorer subjective health status and greater chewing discomfort are associated with a higher likelihood of elevated PHQ-9 scores. Figure 4 illustrates how each feature affected the model's predictions.

### 3.5 Anchor rules derived from the top 15 most important features

To enhance model interpretability, the anchor method was applied. Anchor explanations identify sets of sufficient conditions that locally guarantee high-precision predictions. Table 2 presents the anchor rules extracted at precision thresholds of 0.90 and 0.95.

**Table 2:** Anchor rules identified at different precision thresholds.

**Precision threshold**	**Anchor conditions**	**Precision**	**Coverage**
0.90	Stress recognition > 1.00 and Frequency of contact with neighbors ≤ 1.00	0.93	0.32
0.95	Stress recognition > 1.00 and Frequency of contact with neighbors ≤ 1.00	0.96	0.32

At a precision threshold of 0.90, the anchor condition “Stress recognition >1.00 and Frequency of contact with neighbors ≤ 1.00” was identified, achieving a precision of 0.93 and a coverage of 32%. Increasing the threshold to 0.95 yielded the condition “Stress recognition >1.00 and Frequency of contact with neighbors ≤ 1.00,” with a higher precision of 0.96 and the same coverage. Although increasing the precision threshold typically reduces coverage due to the stricter requirement for prediction consistency, in this study, the coverage remained stable, indicating the robustness and consistency of the identified anchor conditions.

Precision represents the proportion of instances where the model prediction remains consistent when the anchor conditions are satisfied, reflecting the reliability of the explanation. Coverage indicates the proportion of the entire dataset to which the anchor condition applies, providing insight into the generalizability of the rule within the local region.

These results suggest that higher stress recognition combined with lower frequency of contact with neighbors consistently predicts elevated PHQ-9 scores, reinforcing the critical role of psychological and social factors in depression risk.

## 4 Discussion

This study examined key predictors of depression in culturally diverse South Korean families using a finalized ML model and comprehensive variables from the nationally representative KCHS dataset. Unlike prior studies focused on adolescents, our analysis targeted adults aged 19 and older, offering valuable insights for future policy and support strategies.

The top 15 predictors of depression identified through SHAP analysis included factors related to mental health, physical health, personal traits, and social environment. These multidimensional variables provide a comprehensive view of depression risk in culturally diverse families.

We compared the performance of six different models to determine the best fit and interpretability for predicting depression. To prevent overfitting and ensure generalizability, all six machine learning models in this study were trained using five-fold cross-validation. As a result, performance metrics such as *R*^2^, MAE, and RMSE showed minimal differences between the training and test datasets across all models, demonstrating stable and reliable predictive performance without signs of overfitting. Based on these criteria, we selected the XGBoost model and analyzed the importance of each predictive factor using a SHAP bar plot. Generally, boosting models—including XGBoost (known for high accuracy and efficiency)—outperform random forests but may suffer from long training times and overfitting risks (36). However, in our study, the XGBoost model demonstrated stable performance without overfitting. XGBoost uses parallel processing and early stopping to train models quickly and perform optimally regardless of the size of the data. It also uses regularization to limit the growth of the tree that forms the model to prevent overfitting (32). Previous research has identified LightGBM as the most effective model for predicting depression (37, 38). Analytical outcomes derived from ML can vary depending on factors such as the ratio of training to test data and the specific parameter settings of the models. Therefore, standardizing training conditions, as implemented in this study, and conducting cross-validation across multiple models are essential steps to enhance the reliability and generalizability of the results.

The explanatory power of the model in this study (*R*^2^ = 33.1%) is not particularly high in absolute terms, but it is considered meaningful given the complex and multifactorial nature of depression. The model also demonstrates good predictive stability (e.g., MAE, RMSE) and interpretability (e.g., SHAP), making it practical for use. In particular, it has strong applicability for public health interventions and identifying high-risk groups.

The top five predictors of depression in culturally diverse families were stress recognition, experience of extreme sadness or despair, subjective health status, frequency of contact with neighbors, and age, suggesting that mental-health-related factors had the greatest predictive importance. These findings align with a study using a random forest model on KCHS data, which identified stress, gender, occupation, physical activity, and health status as predictors of depression, with stress having the most significant effect (39).

Among mental-health-related factors, stress recognition had the most significant influence on depression, suggesting that stress management education could be incorporated into depression interventions for culturally diverse families, particularly at the family level. Additionally, individuals who had previously experienced extreme sadness or despair were at a higher risk of depression. Therefore, culturally diverse family members with a history of depressive symptoms would benefit from education on symptom management to enhance self-care. Active interventions for stress and depressive symptom management are essential to alleviating depression in this population. The impact of digital media overuse was also significantly associated with depression, consistent with national-scale studies from South Korea, which found that excessive smartphone use was associated with depression (40). Incorporating educational content into digital media habits and depression management could be beneficial.

Regarding physical-health-related factors, less chewing discomfort, a higher weekly frequency of flexibility exercises such as stretching and gymnastics, a higher frequency of handwashing after going out, and better subjective health status were associated with lower levels of depression. A previous study analyzing the relationship between oral health and depression found that depression was significantly more prevalent among individuals experiencing chewing discomfort and recent tooth pain (41). Specifically, depression severity was significantly higher among those with chewing discomfort (42). As chewing discomfort affects food choices, nutrition, social and emotional wellbeing, and overall life satisfaction, it has been linked to negative emotions such as despair, sadness, and depression (42). According to the findings of an ML-based study predicting depression among older adults in China, subjective health status was the strongest predictor of depression, with lower perceived health being associated with higher levels of depressive symptoms. Additionally, a higher frequency of physical activity was associated with lower levels of depression (38). These findings underscore the importance of physical health management, including proper activity and oral health care, in depression prevention for culturally diverse families. It is unlikely that handwashing after going out directly reduces depression; rather, this behavior may reflect greater health consciousness and regular lifestyle habits, which are known to be associated with lower depressive symptoms.

Younger age, a greater self-perception of being thin, lower levels of unmet medical needs in the past year, and more frequent handwashing after going out were associated with lower levels of depression. Among personal factors, the visual explanation does not clearly demonstrate that younger age is associated with lower levels of depression in this study. Prior studies have reported mixed findings regarding the association between age and depression (38). In this study, self-perceived body image—analyzed independently of actual height and weight—was associated with depression such that individuals who perceived themselves as thinner reported lower levels of depression. While one study indicated a stronger association between self-perceived body image and depression than with objectively measured weight, another longitudinal study found no significant link between body image disturbance and later depression (43, 44).

Among social environmental factors, depression levels were higher among culturally diverse families living in urban areas such as Seoul and Gyeonggi but lower among those residing in rural areas. This contrasts with a previous study that reports a 1.09-fold increase in depression among rural residents compared to urban dwellers (17). We speculate that urban environments, characterized by greater competition and limited social exchange, may contribute to this discrepancy, though further research is needed. Additionally, more frequent contact with neighbors and friends were both associated with lower depression levels. This aligns with previous research showing that socially isolated culturally diverse families have 1.36 times higher stress levels, twice the rate of extreme sadness or despair, and 5 times the risk of depression compared to those actively participating in social gatherings (13). Therefore, it is crucial for local authorities to provide spaces for small gatherings, support gathering costs, and offer education to raise awareness about the importance of maintaining social connections. The association between eating breakfast more often per week and reduced depression in this study is consistent with previous findings that eating breakfast no more than twice a week or eating alone is associated with an increased risk of depression (45). Exposure to second-hand smoke in public places was associated with increased depression. These findings support previous findings that second-hand smoke may increase the risk of depression in Korean adults (46). Although the relationship between second-hand smoke exposure and depression remains controversial, previous studies have suggested that second-hand smoke is significantly associated with psychosocial factors that may elevate the risk of depression (47–49). The finding that individuals who have not experienced unmet medical needs in the past year have lower levels of depression aligns with previous research suggesting that unmet medical needs have a positive association with depression (50, 51).

Finally, the anchor method was used to predict depression with the least number of key variables, finding that high stress perception and contact with neighbors once a month or less predicted depression at 93%, indicating that these high-risk groups for depression need active management and policy support, such as interventions to manage stress and improve interpersonal relationships. For instance, to address stress management, it is recommended to implement community-based interventions such as mindfulness-based training programs, cognitive-behavioral stress management workshops, and relaxation techniques. To enhance social connectedness through increased neighbor contact, structured initiatives such as regular community meetings, volunteer engagement programs, and neighborhood buddy systems may serve as effective strategies.

Although the present study reveals important findings, it has several limitations. First and foremost, the model was not validated using a separate, external dataset, primarily due to the practical difficulty of obtaining a comparable national-scale dataset. Therefore, it is necessary to conduct replication studies as new national-level data becomes available. Additionally, the main variables were restricted to those available in the KCHS, and as a cross-sectional survey, the KCHS does not allow for determining causal relationships between depression and its influencing factors. This study has several strengths, including the use of nationally representative data, the application of robust ML methods with explainability techniques, and the identification of a high-precision anchor rule that enables targeted intervention for high-risk groups. However, the anchor rule explained only 32% of the sample, limiting its generalizability. Additionally, the cross-sectional nature of the data prevents causal inference, and important psychosocial variables may have been omitted due to dataset constraints. Future studies should explore broader anchor conditions and replicate findings across different populations to enhance external validity and policy relevance.

Furthermore, ML analysis results can differ based on model parameters and the ratio of training to test data. Therefore, it is essential to conduct replication studies under identical training conditions in different countries using the same variables or alternative datasets. In addition, future studies are encouraged to provide deeper insights by analyzing SHAP values for each variable significantly associated with depression, or by conducting subgroup analyses based on gender, age group, or family composition.

## 5 Conclusions

This study employed ML and data from the 2023 KCHS to predict depression in adults from culturally diverse families. We selected 15 independent variables, including factors of mental and physical health, personal aspects, and the social environment. Among the six ML models tested, the XGBoost model demonstrated the best performance and was used for further analysis. Accordingly, our findings identified stress recognition, the experience of extreme sadness or despair, and the subjective health status of depression. The ML methods employed in this study offer advantages over conventional statistical approaches by effectively capturing non-linear relationships and interactions between variables. Furthermore, by incorporating comprehensive, multidimensional assessment indices, our study provides a broader perspective on depression in culturally diverse families.

We anticipate that our findings will serve as foundational data for education and interventions related to depression in culturally diverse families, thus contributing to more effective mental health support and strategy development.

## Data Availability

The datasets presented in this study can be found in online repositories. The names of the repository/repositories and accession number(s) can be found below: The datasets analyzed during the current study are publicly available from the 2023 Korea Community Health Survey (KCHS) and can be accessed at https://chs.kdca.go.kr/chs/index.do.
