# Supplementary material for: Machine learning prediction of depression in culturally diverse families: Findings from the Korea Community Health Survey
Source: Front Public Health. 2025 Sep 18;13:1666084. doi: 10.3389/fpubh.2025.1666084 (PMC12488461; doi:10.3389/fpubh.2025.1666084)
Supplement: Supplementary file 1 [file Table_1.docx]

Supplementary Table 1: Exploratory data analysis of main variables (N=2,568)

| Category | Variables | Categories | | N (%) | | |
| --- | --- | --- | --- | --- | --- | --- |
| Mental-health-related factors | Stress recognition | Low | | 1,989 (77.5) | | |
|  |  | High | | 579 (22.5) | | |
|  | Experience of extreme sadness or despair | No | | 2,331 (90.8) | | |
|  |  | Yes | | 237 (9.2) | | |
|  | The impact of digital media overuse | Never use | | 399 (15.6) | | |
|  |  | Less than once a month | | 2,013 (78.4) | | |
|  |  | More than a week | | 156 (6.0) | | |
| Physical-health-related factors | Chewing discomfort | Uncomfortable | | 510 (19.8) | | |
|  |  | Neutral | | 365 (14.2) | | |
|  |  | Comfortable | | 1,693 (66.0) | | |
|  | Subjective health status | Poor | | 397 (15.5) | | |
|  |  | Fair | | 1,131 (44.0) | | |
|  |  | Good | | 1,040 (40.5) | | |
|  | Weekly frequency of flexibility exercises | Never | | 1,338 (52.1) | | |
|  |  | 1-3day | | 556 (21.2) | | |
|  |  | 4 or more days | | 674 (26.2) | | |
|  | Handwashing after going out | No | | 59 (2.3) | | |
|  |  | Yes | | 2,509 (97.7) | | |
| Personal factors | Self-perceived body image | Underweight | | 443 (17.2) | | |
|  |  | Normal weight | | 1,157 (45.1) | | |
|  |  | Overweight | | 968 (37.7) | | |
| Social-environmental factors | Area of residence | Seoul/Gyeonggi | | 664 (25.9) | | |
|  |  | Metropolitan city | | 392 (15.3) | | |
|  |  | Rural area | | 1,512 (58.9) | | |
|  | Unmet medical needs in the past year | No | | 2,159 (84.1) | | |
|  |  | Yes | | 173 (6.7) | | |
|  |  | Never needed a doctor's appointment (test or treatment) | | 236 (9.2) | | |
|  | Exposure to secondhand smoke in indoor public places | Yes | | 278 (10.8) | | |
|  |  | No | | 2,290 (89.2) | | |
|  | Weekly frequency of breakfast consumption | Almost never (0 times per week) | | 482 (18.8) | | |
|  |  | 1-4 times per week | | 366 (21.5) | | |
|  |  | 5-7 times per week | | 1,720 (67.0) | | |
|  | Frequency of contact with neighbors | Less than once a month | | 983 (38.3) | | |
|  |  | 2-3 times a month< | | 551 (21.5) | | |
|  |  | 2-3 times a week> | | 1,034 (40.3) | | |
|  | Frequency of contact with friends | Less than once a month | | 598 (23.3) | | |
|  |  | 2-3 times a month< | | 729 (28.4) | | |
|  |  | 2-3 times a week> | | 1,241 (48.3) | | |
|  |  | M ± SD | Min | Max | Skew | Kurt |
| PHQ-9 |  | 11.10 ± 2.96 | 9.0 | 33.0 | 2.68 | 10.37 |
| Personal factors | Age | 50.55 ± 15.72 | 19.0 | 94.0 | 0.29 | -0.34 |

**Note:** Age: Chronological age measured in complete years; Area of residence: A unique identifier representing the administrative province or region; Frequency of contact with neighbors: The frequency with which respondents meet or communicate with the neighbor they interact with most often; Frequency of contact with friends: The frequency with which the respondent meets or contacts their closest friends (excluding neighbors); Handwashing frequency after returning home: The frequency with which respondents washed their hands after returning home from outings during the past week; Stress recognition: Number of individuals reporting high levels of stress in daily life (i.e., answering “very high” or “fairly high”); The experience of extreme sadness or despair: Number of individuals who experienced a depressive episode lasting at least two consecutive weeks in the past year; The impact of digital media overuse: Whether excessive use of digital media (Internet, gaming, and smartphones) adversely affected daily functioning in the past year; Breakfast frequency per week: Number of days per week respondents consumed breakfast during the past year; Self-perceived body shape: Respondents’ subjective appraisal of their current body shape or build; Chewing discomfort: Proportion of individuals reporting difficulties or discomfort when chewing due to dental or oral problems (assessed while wearing dentures, if applicable); Flexibility exercise practice in the past week: Number of days in the last seven days respondents performed flexibility exercises such as stretching or calisthenics; PHQ-9 (Patient Health Questionnaire-9): Sum of scores from PHQ-9 items reflecting the severity of depressive symptoms; Subjective health status: Respondents’ overall perception of their current health, as assessed by the item “In general, how would you rate your health?”; Unmet medical needs in the past year: Whether respondents needed medical examination or treatment during the preceding 12 months but did not receive it; Secondhand smoke exposure in indoor public places: Self-reported exposure to second-hand smoke in indoor public venues (e.g., cinemas, PC rooms, academies, restaurants, cafés).
