# Supplementary material for: Machine learning prediction of depression in culturally diverse families: Findings from the Korea Community Health Survey
Source: Front Public Health. 2025 Sep 18;13:1666084. doi: 10.3389/fpubh.2025.1666084 (PMC12488461; doi:10.3389/fpubh.2025.1666084)
Supplement: Supplementary file 2 [file Table_2.docx]

Supplementary Table 2: Model hyperparameters

| Model | Hyperparameter | Search Range | Selected Value |
| --- | --- | --- | --- |
| Random Forest | n_estimators | [100, 200, 500] | 500 |
|  | max_depth | [None, 10, 20, 30] | 30 |
|  | min_samples_split | [2, 5, 10] | 2 |
|  | min_samples_leaf | [1, 2, 4] | 2 |
|  | max_features | [‘sqrt’, ‘log2’] | ‘sqrt’ |
| Gradient Boosting | n_estimators | [100, 200, 300] | 200 |
|  | learning_rate | [0.01, 0.05, 0.1] | 0.05 |
|  | max_dapth | [3, 5, 7] | 3 |
|  | min_saples_split | [2, 5] | 2 |
|  | subsample | [0.8, 1.0] | 0.8 |
| Extra Trees | n_estimators | [100, 200, 500] | 200 |
|  | max_depth | [None, 10, 20] | 20 |
|  | min_samples_split | [2, 5, 10] | 2 |
|  | min_samples_leaf | [1, 2] | 2 |
|  | max_features | [‘sqrt’, ‘log2’] | ‘sqrt’ |
| AdaBoost | n_estimators | [50, 100, 200] | 50 |
|  | learning_rate | [0.01, 0.1, 1.0] | 0.01 |
|  | loss | [‘linear’, ‘square’, ‘exponential’] | ‘linear’ |
| XGBoost | n_estimators | [100, 200, 300] | 200 |
|  | learning_rate | [0.01, 0.05, 0.1] | 0.05 |
|  | max_dapth | [3, 5, 7] | 3 |
|  | subsample | [0.8, 1.0] | 0.8 |
|  | colsample_bytree | [0.8, 1.0] | 1.0 |
| LightGBM | n_estimators | [50, 100, 150] | 100 |
|  | learning_rate | [0.01, 0.05] | 0.05 |
|  | max_dapth | [5, 10] | 10 |
|  | num_leaves | [15, 31] | 15 |
|  | subsample | [0.8] | 0.8 |
|  | colsample_bytree | [0.8] | 0.8 |
|  | min_data_in_leaf | [10] | 10 |
